# Age-Related Changes in Epilepsy Characteristics and Response to Antiepileptic Treatment in Autism Spectrum Disorders

**DOI:** 10.3390/jpm13071167

**Published:** 2023-07-21

**Authors:** Beliz Su Gundogdu, John Gaitanis, James B. Adams, Daniel A. Rossignol, Richard E. Frye

**Affiliations:** 1 Koç University School of Medicine, 34450 Istanbul, Turkey; bgundogdu17@ku.edu.tr; 2 Department of Neurology and Pediatrics, Hasbro Children’s Hospital, The Warren Alpert Medical School of Brown University, Providence, RI 02903, USA; jgaitanis@lifespan.org; 3 School for Engineering of Matter, Transport and Energy, Arizona State University, Tempe, AZ 85281, USA; jim.adams@asu.edu; 4 Rossignol Medical Center, Aliso Viejo, CA 92656, USA; 5 Autism Discovery and Treatment Foundation, Phoenix, AZ 85050, USA; 6 Rossignol Medical Center, Phoenix, AZ 85050, USA

**Keywords:** antiepileptic drugs, age, autism spectrum disorder, epilepsy, ketogenic diet, mitochondrial dysfunction, modified Atkins diet, neurodevelopmental regression, seizures

## Abstract

Despite the high prevalence of epilepsy in individuals with autism spectrum disorder (ASD), there is little information regarding whether seizure characteristics and treatment effectiveness change across age. Using an online survey, seizure characteristics, effectiveness of antiepileptic treatments, comorbidities, potential etiologies, and ASD diagnosis were collected from individuals with ASD and seizures. We previously reported overall general patterns of treatment effectiveness but did not examine the effect of seizure characteristics or age on antiepileptic treatment effectiveness. Such information would improve the personalized medicine approach to the treatment of seizures in ASD. Survey data from 570 individuals with ASD and clinical seizures were analyzed. Seizure severity (seizure/week) decreased with age of onset of seizures, plateauing in adolescence, with a greater reduction in generalized tonic–clonic (GTC) seizures with age. Seizure severity was worse in those with genetic disorders, neurodevelopmental regression (NDR) and poor sleep maintenance. Carbamazepine and oxcarbazepine were reported to be more effective when seizures started in later childhood, while surgery and the Atkins/modified Atkins Diet (A/MAD) were reported to be more effective when seizures started early in life. A/MAD and the ketogenic diet were reported to be more effective in those with NDR. Interestingly, atypical Landau–Kleffner syndrome was associated with mitochondrial dysfunction and NDR, suggesting a novel syndrome. These interesting findings need to be verified in independent, prospectively collected cohorts, but nonetheless, these data provide insights into novel relationships that may assist in a better understanding of epilepsy in ASD and provide insight into personalizing epilepsy care in ASD.

## 1. Introduction

Autism spectrum disorder (ASD) is a behaviorally defined disorder that is estimated to affect 2% or more of children in the United States [1]. Some of the most challenging aspects in medically managing children with ASD are the medical comorbidities associated with ASD. A recent retrospective analysis of medical claims data suggests that a subgroup of about 24% of children with ASD have an unusually high number of comorbid conditions [2], while other studies report an average of 4–5 comorbidities per child [3]. Comorbidities are wide ranging and include intellectual disability, sensory sensitivity, immune problems such as allergic rhinitis [4], gastrointestinal (GI) abnormalities [5] such as irritable bowel syndrome [4], sleep problems [6], psychiatric conditions such as attention deficit hyperactivity disorder [7] and anxiety [8], and neurological conditions such as seizures and epilepsy [9]. 

Epilepsy is one of the most common comorbid conditions associated with ASD. Individuals with ASD have a 3-to-22-fold increased risk for developing seizures as compared to the typically developing population [10], and clinical studies estimate that seizures co-occur in 25% [11] to 46% [12] of those with ASD. In contrast, only 4–10% of typically developing children will experience a seizure [13], with the majority experiencing febrile seizures, a benign self-resolving disorder [14]. Recent systematic reviews estimate the prevalence of epilepsy in ASD to be 6.3% [15] to 12.1% [16]. This is markedly higher than typically developing children, where the prevalence is about 1% [17]. Furthermore, up to 86% of those with ASD, many of them without clinical seizures, demonstrate interictal epileptiform discharges (IEDs) on electroencephalograph (EEG) [18,19,20], and one study using magnetoencephalography has linked a unique pattern of IEDs to ASD diagnosis [21]. This can be compared to individuals with suspected seizures, where 27% were found to have IEDs on ambulatory EEG [22]. Thus, seizures, epilepsy and IEDs appear to be more prevalent in ASD as compared to the non-ASD population.

Because there are a wide variety of treatments for seizures in ASD, including anti-epileptic drugs (AEDs), nutritional supplements, vitamins, special diets and other alternative medical treatments [18], we previously conducted an online survey where caretakers of individuals with ASD rated the perceived ability of standard and alternative treatment to improve or worsen seizures [19]. The analysis of the 733 respondents demonstrated that, in general, AEDs were more effective at controlling seizures but caused more adverse effects (AEs) on behavior, sleep and cognition, while non-AED treatments were, in general, less effective for controlling seizures but had a positive effect on behavior, sleep and cognition. Further analysis demonstrated that four AEDs, valproic acid, lamotrigine, levetiracetam and ethosuximide, were rated as controlling seizures the best and had the mildest AEs, while four non-AED treatments, the ketogenic diet (KD), the Atkins/modified Atkins diet (A/MAD), the gluten free casein free diet and hyperbaric oxygen therapy, were most beneficial for controlling seizures while having positive effects on behavior, sleep and cognition, with the KD having a markedly more positive effect than the other non-AED treatments. 

Although the results from our previous study were interesting and informative, it only provided an assessment of potential treatments that would be generally helpful. However, as discussed below, ASD has marked heterogeneity which might affect the response to seizure treatment. The influence of individual characteristics of those with ASD has not been investigated. For example, children with ASD who experience neurodevelopmental regression (NDR) [23] have a worse prognosis if the NDR is heralded by a seizure [24]. Thus, in this article, we use the previously collected survey data to examine the effect of important ASD and epilepsy characteristics on response to seizure treatment. 

One of the other possible reasons for the heterogeneity in response to epilepsy treatment in ASD may be the various underlying etiologies, such as metabolic diseases and genetic syndromes, which can cause both seizures/epilepsy and ASD. This is significant as ASD is associated with a high rate of metabolic diseases. For example, 5% of children with ASD have mitochondrial disease, and 30% or more exhibit mitochondrial dysfunction (MD) [25], while cerebral folate deficiency (CFD) is estimated to occur in 38% of those with ASD, with 75% of these demonstrating epilepsy [26]. Additionally, many genetic syndromes associated with ASD are also associated with epilepsy [18], especially those genetic syndromes where gene variants affect synaptic plasticity, ion channels, neurotransmitter receptors and synaptic structure and function [27]. There is also evidence that seizure characteristics are associated with ASD characteristics. 

Furthermore, there is some evidence that the types and severity of seizures may vary with age in ASD [28]. Thus, we hypothesized that the effectiveness of epilepsy treatments varies with age, and we believe this is due to the atypical course of brain development in ASD. For example, epilepsy in ASD can be associated with genetic abnormalities which not uncommonly cause congenital brain malformation such as neuronal migration disorders [29]. Epilepsy in such disorders tends to start early in life [30], although studies do suggest this does depend on the location of the malformation, at least for focal cortical dysplasia [31]. Other data have demonstrated that brain growth is altered in those with ASD [32] with implication of differences in age-dependent neuronal synaptic development [33] and white matter organization [34]. Although many alternations in brain development are believed to occur in early childhood, other studies suggest alteration in brain maturation extends into adolescence [35]. Indeed, a recent study suggests age-dependent dysregulation in GABAergic and glutamate signaling pathways starting in adolescence and extending into adulthood [36]. Thus, given the dynamic changes in brain development across childhood, adolescence and young adulthood, it is likely that different ages may be associated with different seizure characteristics and treatment responses.

Thus, we investigated variations in seizure characteristics, NDR profiles, medical comorbidities, underlying etiologies and treatment effectiveness in those with ASD using our previously collected survey data to examine variations in participant characteristics, particularly with respect to the age of seizure onset. As this survey was conducted in the past, it contains limitations with respect to the drugs and diagnostic criteria at the time. Despite this, we believe these data provide a starting point to understand seizure and epilepsy treatments in ASD, so future prospective studies on seizure treatment in ASD can be designed to look at these variables more critically.

## 2. Materials and Methods

### 2.1. Institutional Review Board Approval

This study was conducted in accordance with the Declaration of Helsinki and the Institutional Review Board of the University of Texas Health Science Center at Houston (the senior author’s primary institution at the time of the study). Since the survey was anonymous and did not contain any unique identifying information or protected health information, the study qualified for category 2 exempt status according to 45 CFR 46.101(b) and did not require full-board review. Information regarding Institutional Review Board approval and contact information was provided on the first page of the survey. The survey did not record any identifying information of the responder, nor did it collect any protected health information identifiers, so data were automatically de-identified for analysis.

### 2.2. Survey

The survey was designed using the principles outlined by Keenan [16] and implemented with the SurveyMethods, Inc. website software (www.SurveyMethods.com (last accessed on 27 October 2009)). The survey was developed based on the consensus of an expert panel as part of the Elias Tembenis Seizure Think Tank specifically focusing on the treatment of seizures in ASD as described in our previous publication [19].

Basic information was collected including age, gender, ASD DSM-IV-TR-based diagnosis and developmental profile, commonly associated comorbid medical conditions including genetic disorders, prematurity, intellectual disability (ID), MD, growth, sleep, GI disorders, allergies and sensory disorders. The DSM-IV-TR was the current version during the data collection period. Since sensory symptoms were not included in the DSM-IV-TR diagnostic criteria, they are considered comorbidities in this study.

Specific information was obtained regarding seizure characteristics. The age at which seizures started was ascertained. Multiple seizure types could be indicated, including generalized tonic–clonic (GTC), focal and absence. In addition, multiple epilepsy syndromes could also be indicated, including typical (LKS) or atypical (aLKS) Landau–Kleffner syndrome, IEDs, Lenox–Gastaut syndrome (LGS) and/or infantile spasms (IS). Seizure severity, as represented by the seizure frequency (seizures per week), was queried for periods in which the seizures were the worst and best controlled. Participants indicated whether the seizures resolved and, if so, the age at which the seizures resolved. If the seizures did not resolve, the patient was considered to have non-resolved epilepsy. Individuals with non-resolved epilepsies did not necessarily have drug-resistant epilepsy but rather were still being treated for epilepsy. For example, some epilepsies take several years to resolve, and an individual may be maintained on the same drug for many years despite a rare breakthrough seizure. 

Following collection of this basic descriptive information, information on the effectiveness of a wide range of treatments was collected. The respondent rated each treatment that the individual with ASD used on a seven-point scale, including the effect on seizures, sleep, communication, behavior, attention and mood. Any AE could also be reported. Treatments were divided into AED treatment, other seizure treatments, dietary therapies, supplements and alternative therapies. 

### 2.3. Participants

An invitation for the online survey was posted on the website and in email newsletters of multiple autism advocacy groups including the Autism Research Institute, Autism Speaks, Autism Society of America and over 30 other local and national ASD support groups for parents of individuals with ASD. A control survey which collected data on children with ASD without seizures was also included in our original study, but it is not used in the analysis for the current study. The number of participants with clinical seizures and seizure characteristic information completed in the survey was 570. 

### 2.4. Statistical Analysis

Ratings were converted into an ordinal scale ranging from 1 to 7 for analysis: substantial negative effect (1), a moderate negative effect (2), a mild negative effect (3), no effect (4), a mild positive effect (5), a moderate positive effect (6), and a substantial positive effect (7). Although the response scale was ordinal, the response distribution was found to be normally distributed, thus allowing the use of parametric analyses for treatment ratings. 

Several variables are used to describe seizure characteristics (see Supplementary Table S1 for a list of patient and seizure characteristics). Non-resolved epilepsy refers to individuals in which seizures were continuing at the time of the survey. Duration of epilepsy refers to the number of years that the child experienced epilepsy and was only relevant in those in which seizures resolved. 

As this study was designed to look at the association among variables, linear regression analysis was felt to be the best method for extracting such associations. Examining combinations of treatments would decrease the number of cases to the extent that a valid analysis could not be performed, so each epilepsy treatment was examined separately.

Other continuous variables were also found to be normally distributed. Continuous dependent variables were analyzed using the generalized linear model with dichotomous variables for categorical factors and covariates for continuous measures. Note that some individuals could have multiple seizure types and multiple ASD diagnoses, so the analysis provided a determination of these specific seizure types and an ASD diagnosis independent of whether other seizure types and ASD diagnosis were present. When independent variables were dichotomous factors, logistic regression was implemented.

The analysis of seizure treatments or patient characteristics was conducted on the data from each seizure treatment or patient characteristic individually. Thus, each treatment represents a separate dataset and the use of linear regression to analyze all factors at once eliminated the need for any post hoc comparisons. Thus, no formal multiple comparison correction was necessary. However, because of the large number of variables being analyzed, alpha was set to *p* ≤ 0.01 to be conservative. 

## 3. Results

The general characteristics of children with clinical seizures overall and for specific epilepsy syndromes are provided in Supplementary Table S1. As seen from this table, most individuals with ASD were male (77.9%), with a mean age of seizure onset in early childhood (5 y 11 m) and a diagnosis of Autistic Disorder (71.7%).

The results are divided into several parts. First, the changes in seizure characteristics across the age of onset were examined. Second, the relationship between specific patient characteristics and seizures was examined. Third, the relation between treatment effectiveness ratings and patient characteristics, including age of onset, was examined. Fourth, rare epilepsy syndromes, specifically IS, LKS, aLKS and LGS, were examined in detail. For our analysis, as stated above, seizure severity is measured as seizures per week when seizures were under the worst and best control.

### 3.1. Seizure Characteristics across the Age Span

#### 3.1.1. Seizure Severity vs. Age of Onset

**Figure 1:**
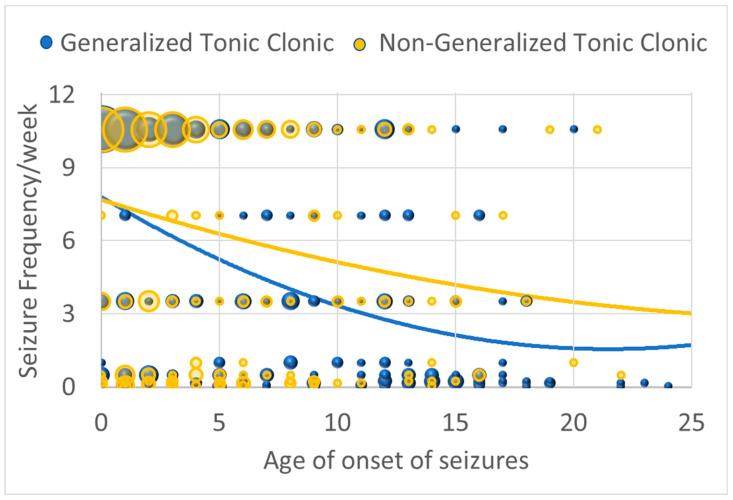
Seizure severity (as indexed by seizure frequency/week when seizures are under the worst control) by age of onset of seizures for those with generalized seizures and those with non-generalized seizures. The size of the circles are proportional to the number of responders with the specific frequency.

Seizure severity when the seizures were under the worst control demonstrated a curvilinear relationship with age of onset such that seizure severity decreased with age of seizure onset, being most severe if starting in childhood and then decreasing and plateaued for those with seizure onset in adolescence [Linear: χ^2^(1) = 17.73, *p* ≤ 0.001; β = −0.434; Curvilinear: χ^2^(1) = 6.70, *p* ≤ 0.01; β = 0.01] (Figure 1). However, for those with GTC seizures, this decrease with age of onset was accentuated [interaction with linear component: χ^2^(1) = 6.65, *p* ≤ 0.01; β = −0.129]. In other words, it appears that GTC and non-GTC seizures are just as likely when seizures start early in life, while non-GTC seizures seem to predominate as the age of seizure onset increases.

For those who experienced NDR (27.7% of the sample), seizure severity when the seizures were under the worst control was more severe by about one seizure per week [χ^2^(1) = 8.95, *p* ≤ 0.01; β = 1.188]. Similarly, for those who experienced absence seizures, seizure frequency was more severe by about one seizure every 4 days [χ^2^(1) = 21.33, *p* ≤ 0.001; β = 1.657].

Seizure severity when the seizures were under the best control was not related to age, but it was worse in those who experienced absence seizures by about one seizure every 4 days [χ^2^(1) = 21.33, *p* ≤ 0.001; β = 1.657] and better in those who experienced GTC seizures by about one seizure every week [χ^2^(1) = 15.39, *p* ≤ 0.001; β = −1.001].

#### 3.1.2. Epilepsy Resolution

Non-resolved epilepsy (17.4% of the sample) was less likely if focal seizures were reported [χ^2^(1) = 13.57, *p* ≤ 0.001; OR = 0.40 (0.24,0.64)].

#### 3.1.3. Duration of Epilepsy

Duration of epilepsy in those children in which epilepsy resolved (82.6% of the sample) was related to ASD diagnosis such that seizures continued 2.7 years and 3.8 years longer in Autistic Disorder and Asperger syndrome, respectively [χ^2^(1) = 7.46, *p* ≤ 0.01; β = 2.68; χ^2^(1) = 7.73, *p* ≤ 0.01; β = 3.80]. Duration was also longer by 2.6 years for those who experienced absence seizures [χ^2^(1) = 10.39, *p* ≤ 0.001; β = 2.63].

### 3.2. Relation between Patient Characteristics and Seizures

#### 3.2.1. Neurodevelopmental Regression in Epilepsy

Risk of NDR increased with seizure frequency when seizures were at their worst control [χ^2^(1) = 7.14, *p* ≤ 0.01; OR 1.06 (1.02. 1.10)]. Seizure severity, seizure resolution, seizure type or ASD diagnosis were not related to any trigger variable.

#### 3.2.2. Potential Underlying Etiology

Age of seizure onset was 1.9 years earlier for those with mitochondrial [χ^2^(1) = 6.18, *p* ≤ 0.01; β = −1.872] disorders.

In those with genetic disorders, seizure severity was more severe by 2.5 seizures per week when seizures were best controlled [χ2(1) = 25.45, *p* ≤ 0.001; β = 2.492] and higher by 3.1 seizures per week when seizures were worst controlled [χ^2^(1) = 16.87, *p* ≤ 0.001; β = 3.099].

GTC seizures were less likely in those with mitochondrial disorders [χ2(1) = 6.43, *p* ≤ 0.01; OR = 0.47 (0.26, 0.84)].

#### 3.2.3. Comorbidities and Epilepsy: General Medical Conditions

The relationship between seizure variables and the general medical conditions of prematurity, cerebral palsy, microcephaly, macrocephaly, ID and growth failure are reported in this section.

Age of seizure onset was 1.7 years later in those with macrocephaly [χ^2^(1) = 6.00, *p* ≤ 0.01; β = 1.790].

Seizure severity when seizures were best controlled was better by 1.7 seizures per week in those with microcephaly [χ^2^(1) = 6.508, *p* ≤ 0.01; β = −1.622].

Seizures were less likely to resolve in those with ID [χ^2^(1) = 7.03, *p* ≤ 0.01; OR = 0.45 (0.25, 0.81)]. In those in which seizures resolved, failure to thrive was associated with 3.0 more years of epilepsy [χ^2^(1) = 6.12, *p* ≤ 0.01; β = 2.967].

GTC seizures were less likely in those born premature [χ^2^(1) = 7.46, *p* ≤ 0.01; OR = 0.52 (0.33, 0.83)] and more likely in those with ID [χ^2^(1) = 4.82, *p* ≤ 0.05; OR = 1.56 (1.05, 2.32)].

Focal seizures were more likely with ID [χ^2^(1) = 10.84, *p* ≤ 0.001; OR = 1.89 (1.30, 2.77)].

#### 3.2.4. Comorbidities and Epilepsy: Sleep

Poor sleep maintenance was associated with a diagnosis of Autistic Disorder [χ^2^(1) = 9.13, *p* ≤ 0.01; OR = 1.80 (1.23, 2.64)] and an increased seizure frequency when seizures were at their best control [χ^2^(1) = 6.72, *p* ≤ 0.01; OR = 1.08 (1.02, 1.14)]. Insomnia, sleep apnea and restless leg syndrome were not associated with seizure variables.

#### 3.2.5. Comorbidities and Epilepsy: Gastrointestinal Symptoms

NDR was more likely with a diagnosis of enteritis [χ^2^(1) = 14.08, *p* ≤ 0.001; OR = 2.38 (1.51, 3.74)], lymphoid nodular hyperplasia [χ^2^(1) = 6.86, *p* ≤ 0.01; OR = 3.56 (1.38, 9.19)], dysbiosis [χ^2^(1) = 15.28, *p* ≤ 0.001; OR = 2.56 (1.60, 4.10)] and GERD [χ^2^(1) = 8.75, *p* ≤ 0.01; OR = 1.90 (1.24, 2.91)]. Constipation was not related to seizure variables or participant characteristics.

#### 3.2.6. Comorbidities and Epilepsy: Sensory

Increase sensitivity to sounds [χ^2^(1) = 7.25, *p* ≤ 0.01; OR = 2.34 (1.26, 4.36)], touch [χ^2^(1) = 18.13, *p* ≤ 0.001; OR = 4.68 (2.30, 9.51)] and smell [χ^2^(1) = 9.06, *p* ≤ 0.01; OR = 3.50 (1.55, 7.93)] was more likely in Asperger syndrome. Decreased touch sensitivity was less likely with older seizure onset [χ^2^(1) = 6.05, *p* ≤ 0.01; OR = 0.83 (0.71, 0.96)]. Decreased sensitivity to taste was more likely in Autistic Disorder [χ^2^(1) = 6.77, *p* ≤ 0.01; OR = 15.06 (1.95, 116.03)] or Asperger syndrome [χ^2^(1) = 14.28, *p* ≤ 0.001; OR = 27.37 (4.92, 152.33)].

#### 3.2.7. Comorbidities and Epilepsy: Allergies

NDR was less likely in individuals in which allergies were reported to affect seizure severity [χ^2^(1) = 7.16, *p* ≤ 0.01; OR = 0.57 (0.38, 0.86)], but specific effects of allergies within each season were not related to seizure variables.

### 3.3. Treatment Effectiveness Ratings

#### 3.3.1. Overall Treatment Effectiveness Ratings

Table 1 provides the overall effectiveness ratings for the perceived effect on seizure control for all treatments. Treatments with average ratings statistically above 4.0 (“no effect”) are provided in bold. Treatments which were statistically above “no effect” and had an average rating of at least “mild” improvement (rating 5 or more) include primidone, valproic acid and levertirecetam for the antiepileptic drugs, surgery and steroids for other seizure treatments, and the KD and A/MAD for diets.

#### 3.3.2. Changes in Effectiveness Rating vs. Age of Onset

**Figure 2:**
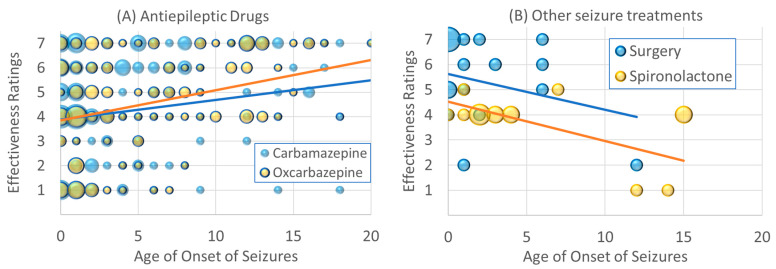

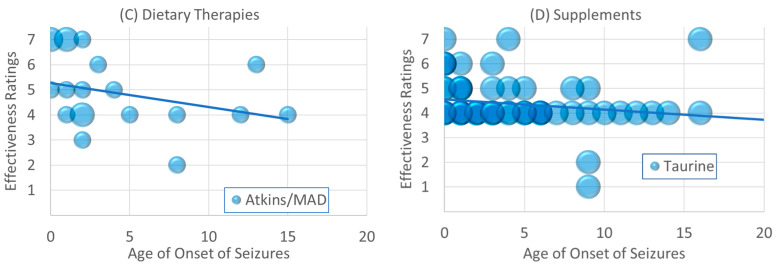
Changes in effectiveness ratings as a function of age of seizure onset for (**A**) antiepileptic drugs, (**B**) other seizure treatments, (**C**) dietary treatments and (**D**) supplements. Size of the circle is proportional to the number of responders with the specific frequency. Please see caption for explanation of the individual colors in each graph.

The effectiveness ratings of carbamazepine [χ^2^(1) = 6.46, *p* ≤ 0.01; β = 0.07] and oxcarbazepine [χ^2^(1) = 19.09, *p* ≤ 0.001; β = 0.15] increased with age of onset. As seen in Figure 2A, for individuals with seizures that start early in life, these AEDs, on average, demonstrated little effectiveness due to the wide variability in the effectiveness ratings. However, for those with seizure onset in late childhood and adolescence, fewer individuals rated these AEDs as negatively affecting seizures, and more rated these AEDs as having a positive effect on seizures.

The effectiveness of surgery [χ^2^(1) = 29.61, *p* ≤ 0.001; β = −0.37] decreased with the age of seizure onset. As seen in Figure 2B, surgery was rated as very effective in those with seizures which started in infancy and younger childhood. The effectiveness of spironolactone decreased with the age of onset of the seizures [χ^2^(1) = 20.68, *p* ≤ 0.001; β = −0.41]. As seen in Figure 2B, for those with seizures that started in early childhood, spironolactone was rated as having no effect, while those with seizures that started in adolescence, spironolactone appeared to have a negative effect.

As seen in Figure 2C, the A/MAD was rated as having an excellent effectiveness in those with seizures which started in infancy and early childhood [χ^2^(1) = 41.61, *p* ≤ 0.001; β = −0.15]. As seen in Figure 2D, Taurine appears to have good effectiveness ratings for those with seizures which started early in life but did not have much of an effect if seizure started after early childhood [χ^2^(1) = 8.89, *p* ≤ 0.01; β = −0.26].

#### 3.3.3. Effectiveness Rating across Seizure Types (GTC, Focal, Absence)

For those with GTC seizures, epilepsy surgery [χ^2^(1) = 10.14, *p* ≤ 0.001] and the A/MAD [χ^2^(1) = 22.71, *p* ≤ 0.001] were rated as 1.89 (0.59) and 1.53 (0.32) points more effective, while valproic acid [χ^2^(1) = 6.00, *p* ≤ 0.01] and steroids [χ^2^(1) = 9.41, *p* ≤ 0.01] were rated as 0.56 (0.23) and 1.25 (0.41) points less effective, as compared to other treatments.

For those with focal seizures, spironolactone [χ^2^(1) = 9.80, *p* ≤ 0.01] was rated as 0.70 (0.22) points more effective, while valproic acid [χ^2^(1) = 8.64, *p* ≤ 0.01] was rated as 0.66 (0.22) points less effective, as compared to other treatments.

For absence seizures, steroids [χ^2^(1) = 6.37, *p* ≤ 0.01] and the KD [χ^2^(1) = 14.35, *p* ≤ 0.001] were rated as 1.03 (0.41) and 1.38 (0.36) points more effective, respectively, while carbamazepine [χ^2^(1) = 17.71, *p* ≤ 0.001], surgery [χ^2^(1) = 7.00, *p* ≤ 0.01] and spironolactone [χ^2^(1) = 65.80, *p* ≤ 0.001] were rated as 1.28 (0.30), 1.57 (0.59) and 1.94 (0.24) points less effective, respectively, as compared to other treatments.

#### 3.3.4. Effectiveness Rating in Non-Resolved Seizures

For those with non-resolved epilepsy, levetiracetam [χ^2^(1) = 8.07, *p* ≤ 0.01] was rated as 1.15 (0.40) points more effective, while phenytoin [χ^2^(1) = 7.80, *p* ≤ 0.01], surgery [χ^2^(1) = 7.42, *p* ≤ 0.01], GFCF diet [χ^2^(1) = 8.50, *p* ≤ 0.01], HBOT [χ^2^(1) = 14.82, *p* ≤ 0.001] and chelation [χ^2^(1) = 7.31, *p* ≤ 0.01] were rated as 1.31 (0.47), 2.61 (0.96), 0.53 (0.18), 1.42 (0.37) and 0.87 (0.25) points less effective, respectively.

#### 3.3.5. Effectiveness Rating across Seizure Severity

For seizure severity, as determined by seizures when they were under the worst control, surgery [χ^2^(1) = 32.07, *p* ≤ 0.001] was rated as 0.82 (0.15) points more effective for each increase in a seizure per week, while zonisamide [χ^2^(1) = 6.14, *p* ≤ 0.01] and A/MAD [χ^2^(1) = 13.72, *p* ≤ 0.001] were rated as 0.13 (0.05) and 0.15 (0.04) points less effective for each increase in a seizure per week.

For seizure severity, as determined by seizures when they were under the best control, valproic acid [χ^2^(1) = 17.49, *p* ≤ 0.001], topiramate [χ^2^(1) = 7.09, *p* ≤ 0.01] and spironolactone [χ^2^(1) = 55.26, *p* ≤ 0.001] were rated as 0.15 (0.04), 0.11 (0.04) and 0.25 (0.03) points less effective for each increase in a seizure per week.

#### 3.3.6. Effectiveness Rating across Autism Diagnosis

Phenytoin [χ^2^(1) = 7.88, *p* ≤ 0.01] was rated as 1.71 (0.61) less effective when the diagnosis included PDD-NOS, while gabapentin [χ^2^(1) = 6.25, *p* ≤ 0.01] was rated as 1.59 (0.63) less effective when the diagnosis included Asperger syndrome.

#### 3.3.7. Effectiveness Rating with Neurodevelopmental Regression

The KD [χ^2^(1) = 10.84, *p* ≤ 0.001] and A/MAD [χ^2^(1) = 48.98, *p* ≤ 0.001] were 1.34 (0.41) and 2.70 (0.39) more effective when the child had a history of NDR.

#### 3.3.8. Effectiveness Rating with Sex

Lamotrigine [χ^2^(1) = 6.50, *p* ≤ 0.01] and spironolactone [χ^2^(1) = 57.18, *p* ≤ 0.001] were 0.80 (0.31) and 2.18 (0.29) points more effective for individuals who were male.

### 3.4. Specific Epilepsy Syndromes

The survey captured patients with several epilepsy syndromes related to epilepsy including IS, LKS, aLKS and LGS. Thus, these groups were examined in detail.

#### 3.4.1. Seizure Variables

Those with LKS were more likely to have a higher seizure severity as measured by seizure frequency at its worst control [χ^2^(1) = 7.57, *p* ≤ 0.01; OR 1.17 (1.05, 1.30)]. IS was less likely as age of seizure onset increased [χ^2^(1) = 15.62, *p* ≤ 0.001; OR 0.38 (0.23, 0.61)].

#### 3.4.2. Neurodevelopmental Regression

Those with aLKS were more likely to have NDR [χ^2^(1) = 9.16, *p* ≤ 0.01; OR 3.53 (1.56, 8.00)].

#### 3.4.3. Potential Underlying Etiology

Those with aLKS were more likely to have a mitochondrial disorder [χ^2^(1) = 7.27, *p* ≤ 0.01; OR 3.54 (1.41. 8.86)], while those with LGS were more likely to have a genetic syndrome [χ^2^(1) = 8.25, *p* ≤ 0.01; OR 5.50 (1.72. 17.58)].

#### 3.4.4. Comorbidities

Those with LGS were more likely to have ID [χ^2^(1) = 7.99, *p* ≤ 0.01; OR 4.97 (1.64. 15.10)].

#### 3.4.5. Effectiveness of Treatments

Clonazepam [χ^2^(1) = 6.65, *p* ≤ 0.01] and zonisamide [χ^2^(1) = 6.06, *p* ≤ 0.01] were rated as 2.49 (0.97) and 2.76 (1.12) points less effective for those with aLKS. Felbamate [χ^2^(1) = 7.41, *p* ≤ 0.01] and A/MAD [χ^2^(1) = 9.44, *p* ≤ 0.01] were rated as 1.74 (0.64) and 1.90 (0.62) points more effective for LGS [χ^2^(1) = 9.44, *p* ≤ 0.01]. L-carnosine [χ^2^(1) = 7.26, *p* ≤ 0.01] was rated as 1.35 (0.50) points better in those with IS.

## 4. Discussion

In this study, we used previously collected survey data to better understand how seizures are related to specific characteristics of individuals with ASD and better understand what factors might affect the effectiveness ratings of treatments for epilepsy.

### 4.1. Seizure Severity Improves with Age

Examining seizure severity across the age of seizure onset demonstrated that seizure frequency was much higher in children with early onset epilepsy, with seizure frequency decreasing significantly throughout childhood and plateauing in adolescence. Seizure severity appeared to be equally severe for those with GTC and non-GTC seizures which started early in life, but the severity of GTC seizures dropped to about half the severity of non-GTC for those that had seizures which started in adolescence. Earlier seizure onset was associated with mitochondrial disorders, while relatively later onset seizures were associated with macrocephaly. Seizure severity was worse in those with genetic disorders, NDR and poor sleep maintenance.

### 4.2. Epilepsy Duration

Focal seizures and ID were associated with epilepsy that was less likely to resolve. In those in which seizures resolved, the number of years that the epilepsy lasted was longer in those that reported an Autistic Disorder or Asperger syndrome diagnosis, failure to thrive, or those that reported absence seizures.

### 4.3. Effectiveness Is Related to Age for Some Treatments

The analysis of seizure treatments demonstrated interesting trends which are consistent with the epilepsy literature and other findings in this study. The data suggest that carbamazepine and oxcarbazepine, medications which primarily treat focal epilepsy, are rated as consistently having a more positive effect in older children and adolescents. Consistently with this, the data from this study also suggest that non-GTC seizures (which include focal seizures) are relatively more common in adolescents. This is a significant finding, in that these medications were clustered in the group that was believed to be the less effective AEDs in our previous study, but the current study demonstrated that this is due to these medications being less effective when seizure start early in childhood in children with ASD. In a recent study examining the benefit on ASD symptoms as compared to AEs, oxcarbazepine was found to have one of the best profiles [37], suggesting that it may be an important agent for controlling seizures in individuals with ASD with late onset seizures.

Surgery and A/MAD treatments appeared to be rated as primarily effective for those with seizures that started early in life. Children with early onset epilepsy tend to have neuronal migration and postmigration organizational abnormalities [30]. Such abnormalities are not uncommonly refractory to medications and may require surgical intervention [38,39]. This could explain why surgery tends to help with epilepsy that starts early in life in individuals with ASD. The A/MAD and KD appeared to be effective treatments, especially for seizures that started early in life, which could be explained by the fact that serious metabolic disorders tend to start early in life. Further definition of this subgroup could indicate those in which dietary interventions should be started early in treatment.

### 4.4. Potential Mechanisms of Low Carbohydrate Diets

The A/MAD has many therapeutic mechanisms. Both the A/MAD and KD are believed to be helpful for treating underlying mitochondrial dysfunction [40], and the data from this study suggest that those with mitochondrial dysfunction have seizure onset earlier in life. This is consistent with the A/MAD being more effective for seizures that start early in life. In addition, the KD and A/MAD were rated as more favorable in those with NDR, another factor which is associated with both classic mitochondrial disease [25,41] and mitochondrial dysfunction [42,43] in ASD. Further, NDR was associated with multiple GI pathological conditions. This is compelling as GI abnormalities are believed to be the result of a disrupted microbiome in ASD [44,45], and some of the therapeutic actions of the A/MAD may occur through normalization of the microbiome [46].

### 4.5. Effectiveness Is Related to Seizure Severity for Some Treatments

Interestingly, several treatments were rated as not effective for more severe seizures. For example, the effectiveness ratings of zonisamide, A/MAD, valproic acid and topiramate were inversely proportional to the seizure frequency, while phenytoin, surgery, GFCF, HBOT and chelation were rated lower in those with epilepsy which did not resolve. The caveat to these findings is that these treatments, such as surgery, are only prescribed when seizures are very severe and refractory, which is also associated with a lower rate of successfully controlling seizures. Thus, the ratings may be biased due to the unique characteristics of individuals to which they were prescribed.

### 4.6. Epilepsy Syndrome May Require Specific Treatments

Analysis of specific epilepsy syndromes revealed some interesting trends. Consistently with known characteristics of syndromes, those with LGS were more likely to have a genetic syndrome. ID and IS were associated with the early onset of seizures. Interestingly, aLKS was associated with both mitochondrial dysfunction and NDR, suggesting that aLKS may be associated with an underlying characteristic of a subset of ASD children with NDR and mitochondrial abnormalities. While the greater effectiveness of Felbamate and A/MAD for LGS is not surprising, the lower-rated effectiveness of Clonazepam and zonisamide for aLKS and higher rating of L-carnosine for IS were novel findings.

### 4.7. Identifying Patient Comorbidities Could Aid Treatment

The findings from this study demonstrate that co-morbidities are related to ASD, suggesting that treatment of such co-morbidities could help with seizure control. For example, poor sleep maintenance was associated with increased seizure severity. Sleep is an important comorbidity to address as an improvement in sleep can improve behavior [6] and quality of life for the whole family [47] as well as improve seizure control [48]. This may be particularly important in ASD as sleep problems are more common in ASD as compared to children with other developmental delays and typically developing children [49].

NDR was also associated with increased seizure severity. Although the reason for NDR in ASD remains enigmatic [50], it could point to specific etiologies associated with NDR which can cause both ASD and epilepsy, such as mitochondrial disease [25], MD [42] and CFD [26] and genetic disorders such as Rett syndrome [51], and channelopathies such as in Dravet syndrome [52]. Metabolic disorders are treatable, so an investigation as to whether they are underlying etiologies in individuals with ASD, NDR and epilepsy may provide effective treatment pathways. This is particularly important in children with ASD, as NDR in those without ASD is very unusual and typically indicated a rare neurodegenerative [53] or metabolic disorder [54] or a rare form of epilepsy such as LKS [55] or electrical status epilepticus during slow wave sleep [56].

### 4.8. Treatments for Epilepsy in Autism Spectrum Disorder

This and other studies [18,19,37] have examined the optimal treatment of epilepsy in children with ASD. In addition to considering the effectiveness of these treatments on seizures, it is important to consider the potential AEs of these medications. A previous study suggested that four AEDs, valproic acid, lamotrigine, levetiracetam and ethosuximide, and four non-AEDs, KD, A/MAD, the gluten free casein free diet and hyperbaric oxygen therapy, were most beneficial for controlling seizures while having minimal AEs. Of course, this needs to be weighed against the common co-morbidities individuals with ASD experience. For example, those with significant GI symptoms or MD should probably avoid valproic acid, while those with growth failure should probably avoid topamax [18].

Children with ASD have idiosyncratic reactions to medications which may be different than those without ASD. A recent study found that lamotrigine, oxcarbazepine and diazepam appear to have the best ratio of improving ASD symptoms as compared to AEs, while valproic acid, levetiracetam, clonazepam and gabapentin had a less favorable ratio with topamax being rated with the least favorable ratio [37].

Lastly, it is important to identify the potential underlying etiology as common metabolic disorders associated with ASD such as mitochondrial disease [25], MD [42] and CFD [26] are treatable, and other rare metabolic disorders that may be amenable to treatment such as urea cycle disorders, succinic semialdehyde dehydrogenase deficiency, creatine deficiency syndrome, biotinidase deficiency, branched-chain ketoacid dehydrogenase deficiency, pyridoxine dependent and responsive seizures, cobalamin metabolism disorders and organic acidurias are associated with ASD and epilepsy [18]. In addition, there are genetic disorders such as tuberous sclerosis, Fragile X and Smith-Lemli–Optiz syndromes which may respond to specific treatments [18].

### 4.9. Study Limitations

This paper had some limitations. Data for this study were collected using an online survey, which might lead to selection bias. Diagnoses of ASD and seizure disorders were not confirmed with standard clinical instruments. The collected data were retrospective in nature. Each treatment was considered in isolation as if it was given in a controlled fashion. It is possible that a caretaker other than the child’s parent filled out the survey. Finally, only 6% of children in the survey had a genetic condition, which may be an undersampling of this group. This study is limited by the self-reported nature of the study and the fact that newer AEDs and ASD treatments were not included in the study.

## 5. Conclusions

This study has revealed some interesting trends in the relationship between seizure onset, age, and ASD characteristics. A better understanding of the factors which modulate epilepsy severity in individuals with ASD will no doubt improve the ability to personalize treatment. Overall, the results suggest that multiple patient characteristics are related to the effectiveness of seizure treatments, and those characteristics may be useful in guiding the choice of treatments for each patient. Future research should focus on defining specific subgroups, if possible, to help better define diagnostic and treatment guidelines for those with ASD.

It is important to note that this paper focused on the effect of treatments on seizure frequency. However, as discussed in detail in our previous paper, AED treatments generally worsen other symptoms, whereas non-AED treatments tend to improve other symptoms. Therefore, it is important to consider both effects on seizure frequency and other symptoms when considering treatments for a patient.

Further prospective clinical studies will be helpful to confirm and expand on these findings. Once these relationships are better understood, it may be possible to design an algorithm for selecting the most promising treatments for children with ASD and epilepsy in a personalized precision fashion in order to optimize outcomes.

## Figures and Tables

**Table 1:** Average (standard error) effectiveness of surveyed treatment for their effects on seizures. Treatments are organized by average effectiveness. Effectiveness ratings statistically greater than 4 (no effect) are bold; effectiveness scale: 1 = substantial negative effect; 4 = no effect; 7 = substantial positive effect.

Antiepileptic Drugs			Other Seizure Treatments			Supplements		
**Treatment**	**N**	**Effectiveness**	**Treatment**	**N**	**Effectiveness**	**Treatment**	N	Effectiveness
**Primidone**	7	**5.29 (0.64)**	**Surgery**	17	**5.41 (0.40)**	**GABA**	47	**4.51 (0.158)**
**Valproic Acid**	247	**5.21 (0.12)**	**Steroids**	33	**5.09 (0.25)**	**Vitamin B6**	82	**4.51 (0.12)**
**Levetiracetam**	151	**5.07 (0.14)**	**VNS**	25	**4.80 (0.34)**	**Magnesium**	96	**4.49 (0.08)**
**Lamotrigine**	171	**4.91 (0.14)**	IVIG	15	4.27 (0.40)	**L-carnosine**	29	**4.41 (0.15)**
**Phenobarbital**	78	**4.90 (0.21)**	Neurofeedback	14	4.21 (0.40)	**Vit B12**	111	**4.37 (0.09)**
**Topiramate**	141	**4.81 (0.13)**	TMS/DCS	2	4.00 (0.00)	**Taurine**	60	**4.35 (0.13)**
**Felbamate**	28	**4.68 (0.33)**	Spironolactone	14	3.71 (0.32)	**DMG**	50	**4.30 (0.125)**
**Zonisamide**	58	**4.67 (0.23)**				**CoQ10**	69	**4.29 (0.08)**
**Carbamazepine**	149	**4.65 (0.16)**	**Diet therapies**			Bacopa	4	4.25 (0.25)
**Oxcarbazepine**	125	**4.59 (0.18)**	**Treatment**	N	Effectiveness	**Glutathione**	66	**4.24 (0.10)**
Tiagabine	7	4.57 (0.30)	**Ketogenic**	48	**5.38 (0.21)**	**L-carnitine**	101	**4.24 (0.09)**
**Phenytoin**	66	**4.52 (0.19)**	**Atkins/MAD**	20	**5.00 (0.33)**	5-HTP	33	4.15 (0.13)
Ethosuximide	27	4.44 (0.32)	**SCD**	34	**4.74 (0.21)**	**Alternative therapies**		
Clonazepam	86	4.41 (0.19)	**GFCF**	154	**4.59 (0.08)**	**Treatment**	**N**	**Effectiveness**
Vigabatrin	10	4.40 (0.50)				**HBOT**	44	**4.39 (0.19)**
Gabapentin	43	3.79 (0.27)				Pioglitazone	6	4.33 (0.21)
						Chelation	62	4.08 (0.16)
						Minocycline	1	4.00

## Data Availability

All data are presented within the article.

## Supplementary Materials

The following supporting information can be downloaded at: https://www.mdpi.com/article/10.3390/jpm13071167/s1, Table S1: Patient Characteristics.
