# Supplementary material for: Age-Related Changes in Epilepsy Characteristics and Response to Antiepileptic Treatment in Autism Spectrum Disorders
Source: J Pers Med. 2023 Jul 21;13(7):1167. doi: 10.3390/jpm13071167 (PMC10381477; doi:10.3390/jpm13071167)
Supplement: Supplementary file 1 [file jpm-13-01167-s001.zip › jpm-2473178-supplementary.pdf]

Table S1. Patient Characteristics.

	All Seizures(N=570)		Generalized (N=315)		Focal (N=253)		Absence (N=266)		LKS (N=24)		Atypical LKS (N=26)		LGS (N=15)		IS (N=29)	
	**Average**	**SEM**	**Average**	**SEM**	**Average**	**SEM**	**Average**	**SEM**	**Average**	**SEM**	**Average**	**SEM**	**Average**	**SEM**	**Average**	**SEM**
**Age of seizure onset**	5.88	0.28	7.04	0.43	4.86	0.32	4.59	0.29	4.25	0.92	3.42	0.62	2.13	0.74	0.48	0.24
	**N**	**%**	**N**	**%**	**N**	**%**	**N**	**%**	**N**	**%**	**N**	**%**	**N**	**%**	**N**	**%**
**Sex** (Male)	444	77.9	253	80.3	186	73.5	205	77.1	19	79.2	22	84.6	11	73.3	24	82.8
**Diagnosis**	**N**	**%**	**N**	**%**	**N**	**%**	**N**	**%**	**N**	**%**	**N**	**%**	**N**	**%**	**N**	**%**
Autism Disorder	409	71.8	240	76.2	180	71.1	203	76.3	19	79.2	19	73.1	13	86.7	19	65.5
PDD-NOS	130	22.8	68	21.6	60	23.7	56	21.1	5	20.8	7	26.9	2	13.3	8	27.6
Asperger’s	59	10.4	27	8.6	29	11.5	22	8.2	1	4.2	1	3.8	0	0	2	6.9
**Comorbidity**	**N**	**%**	**N**	**%**	**N**	**%**	**N**	**%**	**N**	**%**	**N**	**%**	**N**	**%**	**N**	**%**
Prematurity	92	16.1	38	12.1	46	18.2	48	18.0	3	12.5	4	15.4	2	13.3	7	24.1
Cerebral palsy	29	5.1	17	5.4	15	5.9	13	4.9	1	4.2	2	7.7	0	0	4	13.8
Sensory integration disorder	275	48.2	146	46.3	122	48.2	141	53.0	15	62.5	17	65.4	8	53.3	15	51.7
ADHD	159	27.9	79	25.1	76	30.0	88	33.1	7	29.2	6	23.1	2	13.3	5	17.2
Hypotonia	143	25.1	77	24.4	63	24.9	72	27.1	4	16.7	13	50.0	6	40.0	9	31.0
Mitochondrial disease	62	10.9	23	7.3	32	12.6	29	10.9	4	16.7	7	26.9	2	13.3	3	10.3
Enteritis	101	17.7	50	15.9	51	20.2	53	19.9	3	12.5	12	46.2	3	20.0	5	17.2
LNH	20	3.5	12	3.8	12	4.7	10	3.8	0	0	6	23.1	0	0	0	0
Dysbiosis	90	15.8	40	12.7	39	15.4	56	21.1	3	12.5	9	34.6	1	6.7	4	13.8
GERD	130	22.8	65	20.6	61	24.1	58	21.8	3	12.5	11	42.3	6	40.0	8	27.6
Constipation	245	43.0	125	39.7	104	41.1	127	47.7	9	37.5	9	34.6	7	46.7	13	44.8
Growth failure	83	14.6	35	11.1	45	17.8	40	15.0	3	12.5	6	23.1	5	33.3	4	13.8
Accelerated growth	51	8.9	23	7.3	17	6.7	34	12.8	2	8.3	1	3.8	0	0	0	0
Microcephaly	24	4.2	9	2.9	12	4.7	15	5.6	0	0	2	7.7	2	13.3	3	10.3
Macrocephaly	66	11.6	30	9.5	35	13.8	29	10.9	2	8.3	4	15.4	2	13.3	1	3.4
Genetic syndromes	40	7.0	24	7.6	21	8.3	20	7.5	1	4.2	1	3.8	5	33.3	5	17.2
Mental retardation	151	26.4	94	29.8	86	34.0	73	27.4	6	20.8	1	3.8	10	66.7	13	44.8
Insomnia	186	32.6	100	31.7	84	33.2	103	38.7	8	33.3	10	38.5	6	40.0	11	37.9
Disturbed sleep maintenance	267	46.8	140	44.4	118	46.6	137	51.5	11	45.8	16	61.5	8	53.3	14	48.3
Sleep apnea	54	9.5	35	11.1	29	11.5	25	9.4	1	4.2	4	15.4	4	26.7	3	10.3
Restless leg	38	6.7	13	4.1	15	5.9	25	9.4	6	25.0	3	11.5	1	6.7	3	10.3
Renal diseases	11	1.9	3	1.0	6	2.4	7	2.6	0	0	1	3.8	0	0	2	6.9
Cardiovascular diseases	9	1.6	6	1.9	4	1.6	5	1.9	0	0	0	0	2	13.3	0	0
Hematological diseases	5	0.9	0	0	4	1.6	1	0.4	0	0	1	3.8	0	0	0	0
**Regression characteristics**	**N**	**%**	**N**	**%**	**N**	**%**	**N**	**%**	**N**	**%**	**N**	**%**	**N**	**%**	**N**	**%**
NDR	158	27.7	88	27.9	67	26.5	66	24.8	7	29.2	15	57.7	4	26.7	7	24.1
Seizure preceding	57	10.0	32	10.2	27	10.7	23	8.6	5	20.8	2	7.7	2	13.3	5	17.2
Fever preceding	47	8.2	29	9.2	19	7.5	24	9.0	2	8.3	4	15.4	0	0	0	0
Viral illness preceding	32	5.6	19	6.0	14	5.5	16	6.0	0	0	2	7.7	2	13.3	1	3.4
Head trauma preceding	6	1.1	3	1.0	4	1.6	3	1.1	1	4.2	0	0	0	0	0	0
	**Average**	**SEM**	**Average**	**SEM**	**Average**	**SEM**	**Average**	**SEM**	**Average**	**SEM**	**Average**	**SEM**	**Average**	**SEM**	**Average**	**SEM**
Age of initial regression	2.13	0.16	2.14	0.24	2.29	0.28	2.22	0.29	1.93	0.25	1.55	0.16	2.13	0.76	0.99	0.25
Duration (weeks)	29.76	3.48	29.25	4.17	28.30	4.34	25.62	3.55	28.56	9.36	31.54	6.88	25.50	10.89	26.90	12.59
Number of regressions	4.28	0.74	3.88	0.64	4.55	0.86	4.70	0.86	2.33	0.47	3.14	0.42	5.50	2.73	2.57	0.57
Seizure refractoriness	**N**	**%**	**N**	**%**	**N**	**%**	**N**	**%**	**N**	**%**	**N**	**%**	**N**	**%**	**N**	**%**
Seizure resolved	471	82.6	269	85.4	227	89.7	223	83.8	21	87.5	19	73.1	14	93.3	22	75.9
	**Average**	**SEM**	**Average**	**SEM**	**Average**	**SEM**	**Average**	**SEM**	**Average**	**SEM**	**Average**	**SEM**	**Average**	**SEM**	**Average**	**SEM**
Duration in years	3.68	0.44	4.01	0.66	4.63	1.10	5.16	0.84	3.60	1.07	7.13	2.08	2.50	-	7.09	2.14
Frequency (best) (weeks)	1.50	0.13	1.03	0.14	1.55	0.19	2.05	0.22	3.06	0.91	2.19	0.67	4.61	1.35	1.91	0.66

Frequency (worst) (weeks)	5.70	0.20	4.96	0.26	6.51	0.28	6.96	0.28	8.50	0.69	6.80	0.91	10.50	0	9.42	0.53
